# Automated detection and quantification of reverse triggering effort under mechanical ventilation

**DOI:** 10.1186/s13054-020-03387-3

**Published:** 2021-02-15

**Authors:** Tài Pham, Jaume Montanya, Irene Telias, Thomas Piraino, Rudys Magrans, Rémi Coudroy, L. Felipe Damiani, Ricard Mellado Artigas, Matías Madorno, Lluis Blanch, Laurent Brochard, the BEARDS study investigators, Tài Pham, Jaume Montanya, Irene Telias, Thomas Piraino, Rudys Magrans, Rémi Coudroy, L. Felipe Damiani, Ricard Mellado Artigas, Matías Madorno, Lluis Blanch, Laurent Brochard, Cesar Santis, Tommaso Mauri, Elena Spinelli, Giacomo Grasselli, Savino Spadaro, Carlo Alberto Volta, Francesco Mojoli, Dimitris Georgopoulos, Eumorfia Kondili, Stella Soundoulounaki, Tobias Becher, Norbert Weiler, Dirk Schaedler, Oriol Roca, Manel Santafe, Jordi Mancebo, Leo Heunks, Heder de Vries, Chang-Wen Chen, Jian-Xin Zhou, Guang-Qiang Chen, Nuttapol Rittayamai, Norberto Tiribelli, Sebastian Fredes, Ricard Mellado Artigas, Carlos Ferrando Ortolá, François Beloncle, Alain Mercat, J. M. Arnal, J. L. Diehl, A. Demoule, M. Dres, S. Jochmans, J. Chelly, Nicolas Terzi, Claude Guérin, E. Baedorf Kassis, J. Beitler, Davide Chiumello, Erica Ferrari Luca Bolgiaghi, V. Fanelli, J. E. Alphonsine, Arnaud W. Thille, Laurent Papazian

**Affiliations:** 1 Keenan Research Centre for Biomedical Science, Li Ka Shing Knowledge Institute, St. Michael’s Hospital, 30 Bond St, Toronto, ON M5B 1W8 Canada; 2 Interdepartmental Division of Critical Care Medicine, University of Toronto, 209 Victoria St, Toronto, ON M5B 1T8 Canada; 3 Université Paris-Saclay, AP-HP, Service de médecine intensive-réanimation, Hôpital de Bicêtre, DMU CORREVE, FHU SEPSIS, Groupe de recherche clinique CARMAS, Le Kremlin-Bicêtre, France; 4 Better Care SL, Sabadell, Spain; 5 Division of Respirology, Department of Medicine, University Health Network, Toronto, Canada; 6 Sinai Health System, Toronto, Canada; 7 St. Michael’s Hospital, Unity Health Toronto, Toronto, Canada; 8 Division of Critical Care, Department of Anesthesia, McMaster University, Hamilton, Canada; 9 Médecine Intensive Réanimation, CHU de Poitiers, Poitiers, France; 10 INSERM CIC 1402, Groupe ALIVE, Université de Poitiers, Poitiers, France; 11 Departamento Ciencias de la Salud, Carrera de Kinesiología, Faculdad de Medicina, Pontificia Universidad Católica de Chile, Santiago, Chile; 12 Surgical ICU, Department of Anesthesia, Hospital Clínic, Barcelona, Spain; 13 Instituto Tecnológico de Buenos Aires (ITBA), Buenos Aires, Argentina; 14 Critical Care Center, Hospital Universitari Parc Taulí, Institut D’Investigació I Innovació Parc Taulí I3PT, Universitat Autònoma de Barcelona, Sabadell, Spain; 15 Biomedical Research Networking Center in Respiratory Disease (CIBERES), Instituto de Salud Carlos III, Madrid, Spain; 16 Saint Michael’s Hospital, Toronto, ON Canada; 17 Dipartimento di Anestesia, Rianimazione ed Emergenza-Urgenza, Fondazione IRCCS Ca’ Granda Ospedale Maggiore Policlinico, Università degli studi di Milano, Milan, Italy; 18 Department of Morphology, Surgery and Experimental Medicine, Intensive Care Unit University of Ferrara, Sant’Anna Hospital, Ferrara, Italy; 19 Anesthesia and Intensive Care, Fondazione Istituto Di Ricovero E Cura a Carattere Scientifico, Policlinico San Matteo, Pavia, Italy; 20 Department of Intensive Care Medicine, School of Medicine, University Hospital of Heraklion, University of Crete, Heraklion, Crete, Greece; 21 Department of Anesthesiology and Intensive Care Medicine, University Medical Center Schleswig-Holstein, Campus Kiel, Kiel, Germany; 22 Critical Care Department, Vall D’Hebron University Hospital, Vall D’Hebron Research Institute and Ciber Enfermedades Respiratorias, Instituto de Salud Carlos III, Madrid, Spain; 23 Intensive Care Medicine, Hospital de Sant Pau, Barcelona, Spain; 24 Department of Intensive Care Medicine, Amsterdam UMC, Amsterdam, The Netherlands; 25 College of Medicine, National Cheng Kung University Hospital, National Cheng-Kung University, Tainan, Taiwan; 26 Department of Critical Care Medicine, Beijing Tiantan Hospital, Capital Medical University, Beijing, China; 27 Division of Respiratory Diseases and Tuberculosis, Faculty of Medicine Siriraj Hospital, Mahidol University, Bangkok, Thailand; 28 Complejo Médico de La Policía Federal Argentina Churruca Visca, Buenos Aires, Argentina; 29 Sanatorio de La Trinidad Mitre, Buenos Aires, Argentina; 30 Hospital Clinic, Barcelona, Spain; 31 Medical Intensive Care Unit, University Hospital of Angers, Angers, France; 32 Service de Réanimation Polyvalente, Hôpital Sainte Musse, Toulon, France; 33 Medical Intensive Care Unit, Hôpital Européen Georges Pompidou Assistance Publique-Hôpitaux de Paris, Paris, France; 34 AP-HP, Groupe Hospitalier Pitié-Salpêtrière Charles Foix, Service de Pneumologie, Médecine Intensive - Réanimation (Département ‘R3S’), Paris, France; 35 Groupe Hospitalier Sud Ile-De-France, Centre Hospitalier de Melun, Melun, France; 36 Médecine Intensive Réanimation, C.H.U de Grenoble-Alpes, Grenoble, France; 37 Division of Pulmonary and Critical Care, Beth Israel Deaconess Medical Center, Massachusetts General Hospital, Harvard Medical School, Boston, MA USA; 38 Division of Pulmonary, Allergy, and Critical Care Medicine, Columbia University College of Physicians and Surgeons, NewYork-Presbyterian Hospital, New York, NY USA; 39 Department of Surgical Science, Division of Anesthesia and Critical Care Medicine, University of Turin, AOU Città della Salute e della Scienza di Torino, Turin, Italy; 40 Médecine Intensive Réanimation, Centre Hospitalier Universitaire de Poitiers, Poitiers, France; 41 Médecine Intensive Réanimation, Hôpital Nord, Hôpitaux de Marseille, Chemin des Bourrely, 13015 Marseille, France

**Keywords:** Reverse triggering, Dyssynchrony, Mechanical ventilation, Lung and diaphragm protection, Respiratory muscles

## Abstract

**Background:**

Reverse triggering (RT) is a dyssynchrony defined by a respiratory muscle contraction following a passive mechanical insufflation. It is potentially harmful for the lung and the diaphragm, but its detection is challenging. Magnitude of effort generated by RT is currently unknown. Our objective was to validate supervised methods for automatic detection of RT using only airway pressure (Paw) and flow. A secondary objective was to describe the magnitude of the efforts generated during RT.

**Methods:**

We developed algorithms for detection of RT using Paw and flow waveforms. Experts having Paw, flow and esophageal pressure (Pes) assessed automatic detection accuracy by comparison against visual assessment. Muscular pressure (Pmus) was measured from Pes during RT, triggered breaths and ineffective efforts.

**Results:**

Tracings from 20 hypoxemic patients were used (mean age 65 ± 12 years, 65% male, ICU survival 75%). RT was present in 24% of the breaths ranging from 0 (patients paralyzed or in pressure support ventilation) to 93.3%. Automatic detection accuracy was 95.5%: sensitivity 83.1%, specificity 99.4%, positive predictive value 97.6%, negative predictive value 95.0% and kappa index of 0.87. Pmus of RT ranged from 1.3 to 36.8 cmH_2_0, with a median of 8.7 cmH_2_0. RT with breath stacking had the highest levels of Pmus, and RTs with no breath stacking were of similar magnitude than pressure support breaths.

**Conclusion:**

An automated detection tool using airway pressure and flow can diagnose reverse triggering with excellent accuracy. RT generates a median Pmus of 9 cmH_2_O with important variability between and within patients.

**Trial registration:**

BEARDS, NCT03447288.
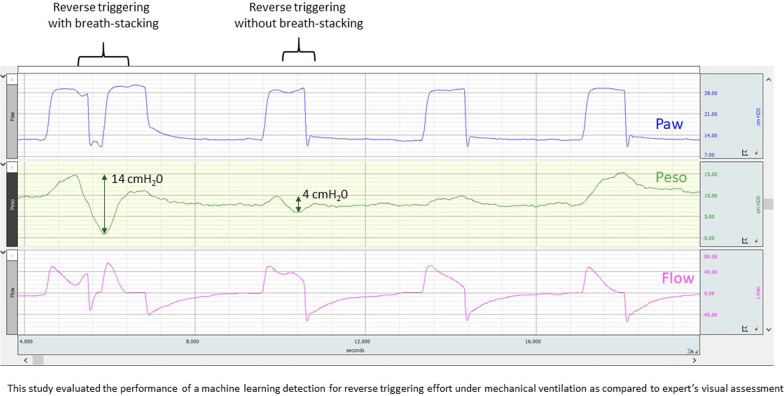

**Supplementary Information:**

The online version contains supplementary material available at 10.1186/s13054-020-03387-3.

## Background

Patient–ventilator dyssynchrony is often associated with poor patient-centered outcomes such as duration of mechanical ventilation or mortality [1–4]. The causality has not been demonstrated, and it is not clear yet whether and when some types of dyssynchrony can directly cause harm or discomfort, or whether others are simply markers of suboptimal ventilator settings or more severe underlying conditions. Poor patient–ventilator interaction is, however, a major reason for administering sedation in the ICU, and therefore this phenomenon deserves attention and a more granular description than referring to dyssynchrony in general [5]. Of major interest, reverse triggering (RT) is a specific form of dyssynchrony defined by the presence of a respiratory muscle contraction following a passive mechanical insufflation as if the contraction was “triggered by” the ventilator [6]. It has been described in intubated patients receiving sedation under controlled ventilation and seems to be very frequent [7–15]. This phenomenon might constitute a regular entrainment (phase locking) of the respiratory rhythm to periodic insufflation, as described in animals [16, 17] and healthy humans [18], but it may also be more irregular and can even occur in brain-dead patients [10]. When the effort generated is strong enough, it induces breath stacking, often misinterpreted to be caused by double triggering (in which the same patient’s inspiratory effort would trigger the first and second mechanical insufflation). Reverse triggering could impact patients’ outcomes through several mechanisms, such as increased tidal volume during inspiration, breath stacking, or through pendelluft during the inspiratory phase [14]. On the one hand, it can generate diaphragm injury when generating strong eccentric contractions during exhalation [19], but on the other hand, when small, diaphragmatic contractions related to RT could be beneficial by preventing muscle disuse and atrophy in sedated patients.

Detection of dyssynchrony in general and RT in particular is challenging requiring additional physiological signals and/or careful attention to the waveforms on the ventilator screens and expertise to properly interpret the signals. Additionally, to have an estimate of the real burden of dyssynchrony 24/7 inspection of the waveforms would be required [20, 21]. The reference technique to detect respiratory muscle activity and accurately diagnose dyssynchrony needs an esophageal catheter or a catheter that captures the electrical activity of the diaphragm (EAdi). Preliminary data suggest a high incidence of frequent RT (> 30%) in patients under assist-control ventilation [22]. Automatic machine learning techniques that do not require visual inspection are needed for understanding the phenomenon and helping the clinician to optimize patient–ventilator interactions [23]. As the first step of a prospective multicenter observational study that aims at establishing the incidence, determinants and consequences of various dyssynchronies during early acute hypoxemic respiratory failure (BEARDS, NCT03447288), we developed and validated an automated algorithm to detect RT only from ventilator signals, i.e., airway pressure and flow. Some of the authors (JM, RM, LBl) had previous experience in developing a dedicated software application to detect other types of dyssynchrony (https://bettercare.es/); this platform was used as a starting point for the current algorithm. In a pre-validation phase, we created, developed and tested algorithms for RT based on airway pressure (Paw) and flow (Additional file 1). Our main objective was to validate the automatic detection of RT by the software using only Paw and flow against a visual assessment of the same tracings by experts having Paw, flow and esophageal pressure (Pes). A secondary objective was to describe the magnitude of the efforts generated during RT.

## Methods

### Definitions

**Fig. 1:**
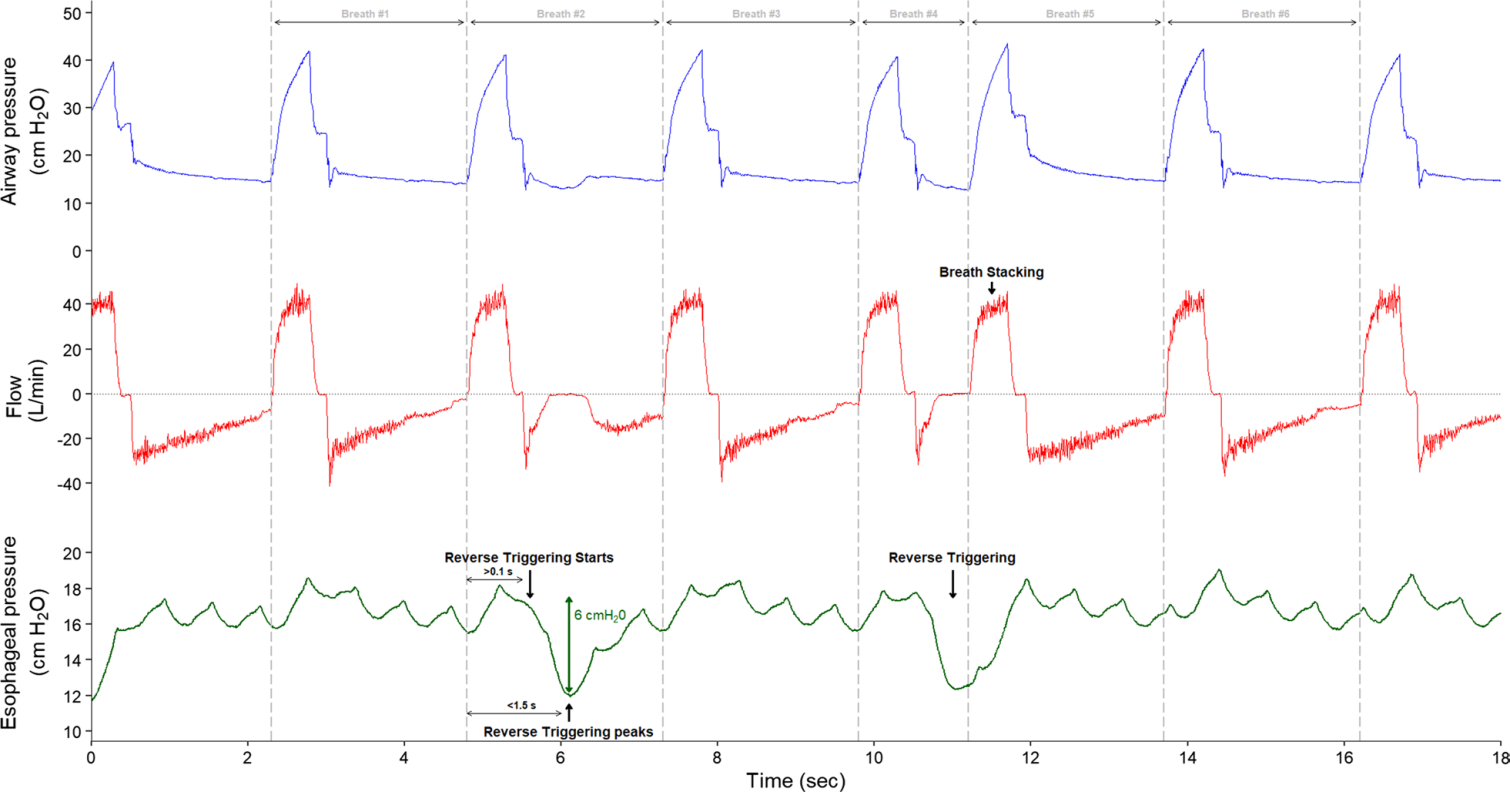
Breath count and events classification methods. This figure shows an example of tracing with breath counts, reverse triggering and breath stacking. Top waveform (blue): airway pressure (Paw); middle waveform (red): flow; bottom waveform (green):  esophageal pressure (Pes). A breath is an insufflation followed by an expiration (even if short and incomplete); nine breaths are present on the figure. There are two reverse triggering on this figure, and the second reverse triggering causes breath stacking (definitions in the text).

*A breath* was defined as an insufflation followed by an expiration, even if short and incomplete. A respiratory cycle started at the beginning of an insufflation and ended at the beginning of the next insufflation as shown in Fig. 1. If an early second insufflation happened before complete exhalation, we counted it as a different breath (and labeled it as a stacked breath).

A *machine-triggered breath* was characterized by the absence of patient’s effort before the machine insufflation.

*Reverse triggering* was defined as an active contraction of the respiratory muscles including the diaphragm (using esophageal pressure or EAdi) starting more than 0.1 s after the start of a machine-triggered insufflation (Fig. 1); also, the maximal Pes deflection (or the EAdi peak) had to happen less than 1.5 s after the machine insufflation had started. Although the effort starts during insufflation, the peak of the effort can still be during insufflation or plateau but often takes place during early expiration [e-Fig. 1 and e-Fig. 2]. We did not incorporate any element of regular entrainment as we have observed multiple examples where a true entrainment is disrupted and becomes complex to recognize. We initially used recordings either with EAdi or esophageal pressure tracings to develop the algorithms, and only tracings with Pes for the validation. We defined a minimal effort before machine insufflation to be considered a patient triggered breath as being a Pes drop of more than 2 cmH_2_0 as compared to baseline or an EAdi > 0.5 µV above the baseline with a Paw drop > 0.3 cmH_2_0. For each tracing, the rate of RT was defined as the following: $\frac{sum\; of\; all\; RTs}{sum\; of\; all\; breaths}.$

*Breath stacking* was the occurrence of a second insufflation before complete exhalation of the volume insufflated in the preceding breath (Fig. 1 and Additional file 1 (e-Fig. 3)).

An active contraction of the inspiratory muscles (Pes negative swing) during the late expiration phase of a patient-triggered breath and not able to trigger a breath defined an *Ineffective effort during expiration*. If this happened after a machine-triggered inspiration, the maximal peak deflection had to occur more than 1.5 s after the machine insufflation to differentiate ineffective effort from reverse triggering.

### Algorithm for detection of reverse triggering

**Fig. 2:**
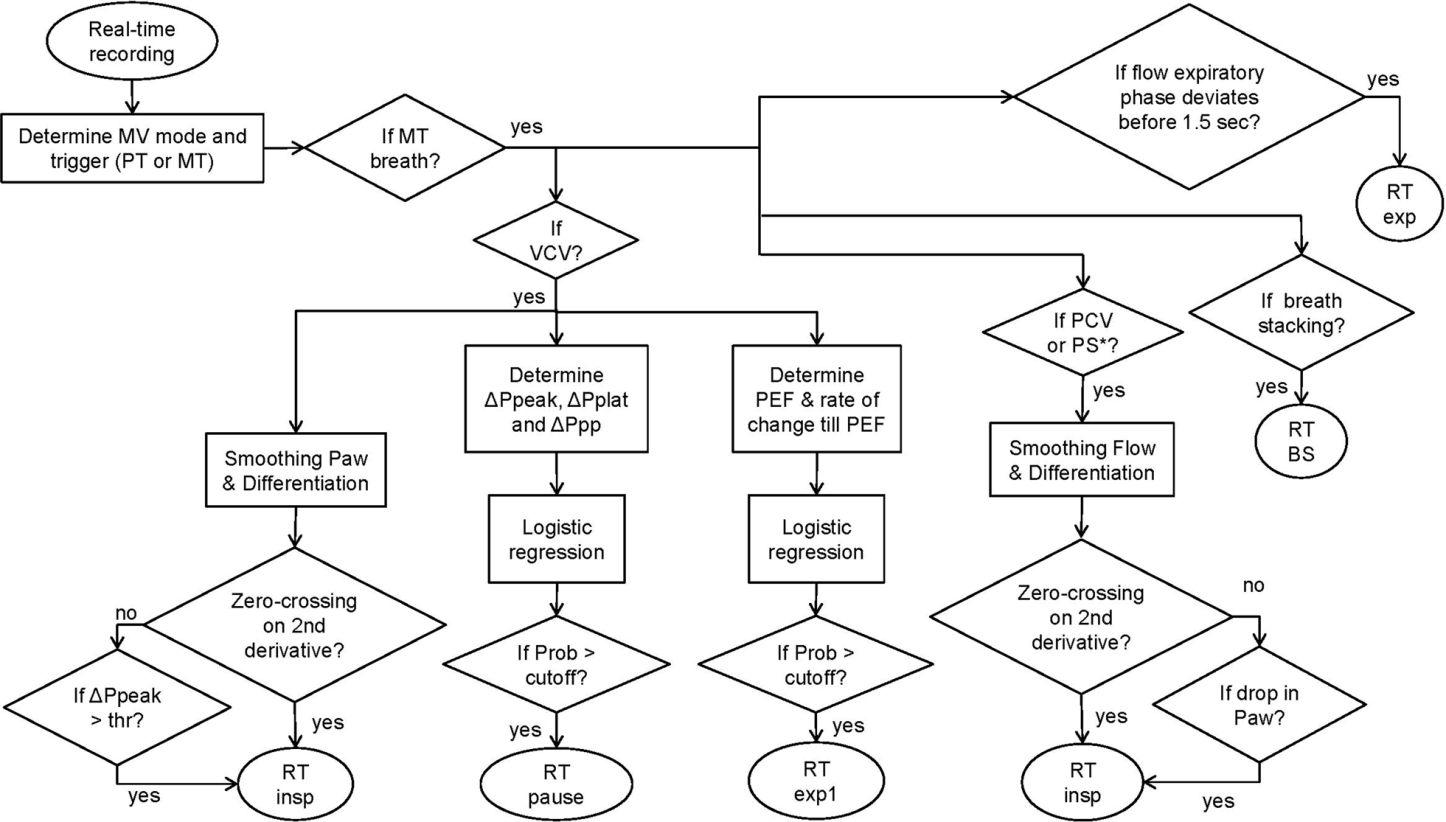
Diagram of the diverse methods implemented for RT events detection. This figure shows the step-by-step approach of the automatic detection algorithm to detect reverse triggering according to the mode of mechanical ventilation and the time of the RT occurrence. *MV* = mechanical ventilation; PT = patient-triggered breath; MT = machine-triggered breath; VCV = volume-controlled ventilation; PCV = pressure-controlled ventilation; PS = pressure support; Paw = airway pressure; ∆Ppeak = variation between the current peak pressure (Ppeak) value and the average of the most immediate previous Ppeak values; ∆Pplat = variation between the current plateau pressure (Pplat) value and the average of the most immediate previous Pplat values; ∆Ppp = difference between ∆Ppeak and ∆Pplat values; PEF = peak expiratory flow; Prob = RT probability values predicted by the logistic regression model; cutoff = optimal probability value for RT event classification; RT insp = reverse triggering during inspiration in VCV and PCV modes, and (*) in auto-triggered breaths in PS; RT pause = reverse triggering during the pause; RT exp1 = reverse triggering late at inspiration producing airflow deformation at the onset of exhalation; RT exp = reverse triggering during expiration occurring within 1.5 s after controlled inspirations; RT BS = reverse triggering producing breath stacking.

Several methods of digital signal processing and supervised machine learning have been combined to detect RT events from flow and Paw waveforms depending on the mode of mechanical ventilation and the phase of the respiratory cycle where the event occurred. These methods intended to account for the different ways RT impacts on either or  both waveforms. For instance, during constant flow, the impact during inspiration will be mainly on Paw, while during pressure controlled ventilation the impact will be both on Paw and on flow. In both situations there may also be an impact on expiratory flow. The signals were smoothed to avoid artifactual detections. The mechanism that initiated the respiratory cycle was automatically detected (i.e., machine/time or patient triggered). The strategies implemented are summarized in Fig. 2, and the details regarding the algorithm are given in the Additional file 1.

### Validation phase

A training set was based on 20 to 40 minutes long tracings displaying visually detectable RT; these tracings mostly came from a previous study for 9 recordings (DIVIP, NCT02434016) and 3 from the BEARDS study. The final validation dataset was only based on recordings from the BEARDS study (and with no patient used in the training dataset). It comprised 20 tracings from 20 different patients among the first 109 patients enrolled in the BEARDS study and the 1087 tracings available: 4 tracings without visible RT, 4 with a low visible rate of RT, 4 with a moderate rate of RT, 4 with a high rate of RT and 4 on PSV. All patients met the criteria for moderate or severe hypoxemic respiratory failure (PaO2/FiO2 < 200 mmHg and deep sedation at inclusion) with unilateral injury or ARDS. Ventilator waveforms and esophageal pressure were synchronously recorded over the first 7 days of mechanical ventilation. We selected 20 patients in order to represent a full spectrum of RT occurrence. A semiquantitative analysis allowed to classify the tracings available (in volume or pressure assist-control mode) according to the estimated frequency of RT: (1) no visible RT; (2) low rate of detectable RT: less than 4 RTs per minute; (3) moderate rate of RT: between 4 and 10 RTs per minute; (4) high rate of RTs: more than 10 RTs per minute. We discarded tracings with technical issues (excessive cardiac artifacts, leaks, uncertain calibration procedure, oscillations impairing the tracing assessment) and randomly selected 4 tracings in each category for the validation. For the 4 tracings with no RT, we purposely selected recordings collected when the patient was receiving neuromuscular blocking agents. Additionally, we randomly selected 4 tracings from the 246 recordings collected while patients were ventilated in pressure support ventilation (PSV). Tracings collected during paralysis or PSV acted as a control to ensure that the algorithm would not have false positives: RT should not occur in these situations because there is either no diaphragm contraction (paralysis) or because the patient triggers all the breaths and there is no passive insufflation (PSV).

Two researchers (TPh and TPi) visually reviewed 10 min of each of these 20 selected tracings and categorized each breath according to the type of trigger (machine vs patient), the presence or absence of RT and the presence or absence of a stacked breath (early additional breath during the exhalation phase) using Paw, flow and Pes. This assessment was used as the gold standard for breaths classification.

Independently, and without the Pes signal, the same breaths were analyzed by the automatic detection software. The results of the visual and automatic detections were merged on a spreadsheet to review agreement and discrepancies breath by breath. All discrepancies were reviewed by a third researcher (RC) blinded to the results obtained with visual or automatic detection. This step confirmed or infirmed the discrepancies.

### Quantification of effort during reverse triggering

One important question regarding the potential risk of RT (for the lung or diaphragm) relates to the amplitude of efforts generated by RT. Therefore, we calculated the muscular pressure (Pmus) of each breath to estimate the range of effort generated during RT and compare it to the Pmus generated during pressure support ventilation [24]. We aimed at estimating the range of effort that generates a deformation of flow and Paw waveforms, as detected by our algorithm. Pmus was calculated as the maximum difference between the Pes and the chest wall relaxation curve according to volume. The latter was calculated using the chest wall compliance measured during passive breaths in the same patient. The FluxMed® software was used for this calculation. Inspiratory efforts with artifact in the Pes signal that prevented from accurate measurement of Pmus were discarded (N = 34 in tracings during assist-control and N = 14 in tracings during pressure support).

### Statistics

Agreement between researchers and automatic detection for the diagnosis of RT was determined with the Kappa statistic [25, 26]. True positives were breaths considered as RT both by visual assessment and by automatic detection. True negatives were breaths considered as not being RT both by visual assessment and by automatic detection. False positives were breaths considered as RT by automatic detection but not by visual assessment. False negatives were breaths considered as RT by visual assessment but not by automatic detection. Standard formulas were used to calculate sensitivity, specificity, positive predictive value (PPV) and negative predictive value (NPV) [27, 28]. Generalized estimating equation was used to compare the magnitude of inspiratory effort for different types of breaths considering each patient a cluster.

Sample size calculation: Our primary objective was to validate the automatic detection versus visual assessment of RT aiming at a Kappa above 0.8 that can be considered an almost perfect agreement. To obtain a kappa of 0.85 with a 95% confidence interval [0.83;0.87] and assuming a rate of RT of 20% with an alpha risk of 0.05, 4655 breaths were estimated to be necessary [25]. Using 20 recordings at a respiratory rate of 20/min, the analysis of 10 min would provide approximately 5000 breaths.

## Results

### Patients and tracings characteristics

The 109 patients included in the BEARDS study had a median [IQR] of 12 [9;15] tracings collected and 58 (53%) of the patients had at least one of their tracings displaying reverse triggering. Tracings from 20 of these patients were randomly selected for the validation (e-Fig. 4). The main characteristics of these patients are presented in Table 1. The tracings used for this validation study were collected at a median [IQR] of 3 [2;4] days from inclusion. Among the 20 tracings selected, 9 (45%) were in pressure assist-control, 7 (35%) in volume assist-control, 4 (20%) in pressure support ventilation mode and 4 were collected in paralyzed patients. At the time of recording, 8 (40%) patients were receiving a vasopressor, 12 (60%) patients were sedated with midazolam, 4 (20%) with propofol, 17 (85%) were receiving an opioid infusion and patients had a median [IQR] Richmond Agitation Sedation Scale [29] of − 4 [− 5;− 2].

**Table 1:** Patients characteristics. Categorial variables are expressed as N (%) and numerical variables as mean ± SD

	N = 20
Female Gender	7 (35.0%)
Age, years	65 ± 12
Height, cm	168 ± 10
Weight, kg	79 ± 30
Comorbidities	
Hypertension	10 (50.0%)
COPD	4 (20.0%)
Diabetes	6 (30.0%)
Chronic kidney disease	4 (20.0%)
Chronic cardiac failure	3 (15.0%)
Hematologic malignancy	1 (5.0%)
Immunosuppression	1 (5.0%)
Chronic liver disease	2 (10.0%)
Cause for intubation	
Hypoxemia	18 (90.0%)
Hypercapnia	1 (5.0%)
Shock	2 (10.0%)
Cardiac arrest	2 (10.0%)
Surgery	1 (5.0%)
Risk factor for ARDS	
Pneumonia	17 (85.0%)
Aspiration	2 (10.0%)
Pulmonary contusion	1 (5.0%)
Non-pulmonary sepsis	1 (5.0%)
Severity at inclusion	
APACHE III	82 ± 29
SOFA	9.5 ± 4.0
pH	7.34 ± 0.09
PaO_2_/FiO_2_ ratio, mmHg	148 ± 42
PaCO_2_, mmHg	45 ± 10
Outcomes	
Discharged alive from the ICU	15 (75.0%)
Discharged alive from the hospital	11 (55.0%)

### Accuracy of reverse triggering detection

A total of 4509 breaths from the 20 recordings were assessed, and 1073 (24%) were considered as RT as per gold standard (visual assessment); almost all RTs (N = 1070) occurred during controlled ventilation and only 3 following auto-triggered breaths in PSV. Among patients in assisted-control mode presenting at least one RT, the rate of RT ranged from 14.8% to 93.3%. The confusion matrix between visual assessment and the automatic detection is shown in Table 2, and the ROC curve is displayed in the Additional file 1 (e-Fig. 5): There were 20% true positive, 76% true negative, 4% false negative and 0.5% false positive. Therefore, automatic detection achieved a global accuracy of 95.5%, sensitivity of 83.1%, specificity of 99.3%, a positive predictive value of 97.6%, a negative predictive value of 94.9% and kappa index of 86.9 [85.1;88.7]. At a tracing level, RT rates (expressed as a percentage of all ventilator breaths) ranged from 0 (patients paralyzed or in PSV) to 93.4%; the median [IQR] was 17.0% [0%;43.3%]. Individually, the accuracy ranged from 73.8 to 100% (median [IQR] 98.1% [95.5%; 100%]). A low accuracy (73.8%) was observed in one patient with numerous but very weak reverse triggering efforts (magnitude ~ 3–4 cm H_2_O) frequently not strong enough to induce a visible deformation on Paw or Flow; this patient had been sedated and had no sign of wakefulness.

**Table 2:** Agreement matrix

	Visual assessment		Total
	RT	No RT	
**Automatic detection**			
**RT**	892	22	914
**No RT**	181	3414	3595
**Total**	1073	3436	4509

### Range of patients’ effort corresponding to RT

**Fig. 3:**
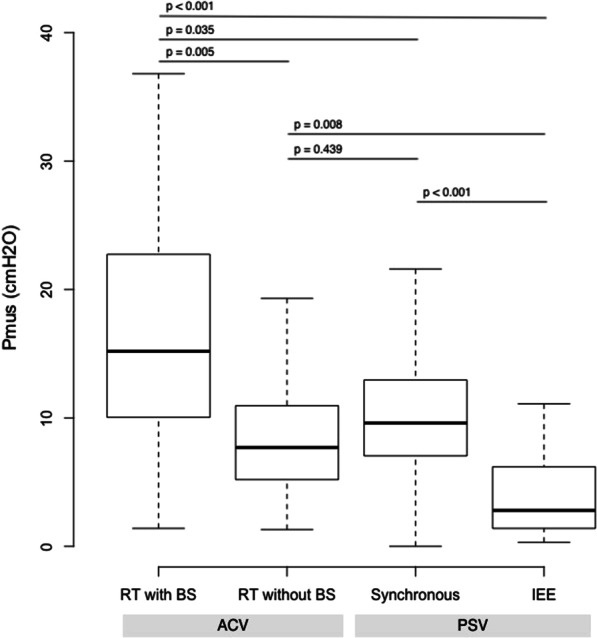
Amplitude of Pmus generated during RT with breath stacking (RT with BS N = 206), without breath stacking (N = 841), during efforts triggering the ventilator in pressure support ventilation (synchronous, N = 682) and during ineffective efforts during expiration (IEE, N = 33). RT = reverse triggering, BS = breath stacking, IEE = ineffective efforts during expiration, ACV = assist-control ventilation, PSV = pressure support ventilation

**Fig. 4:**
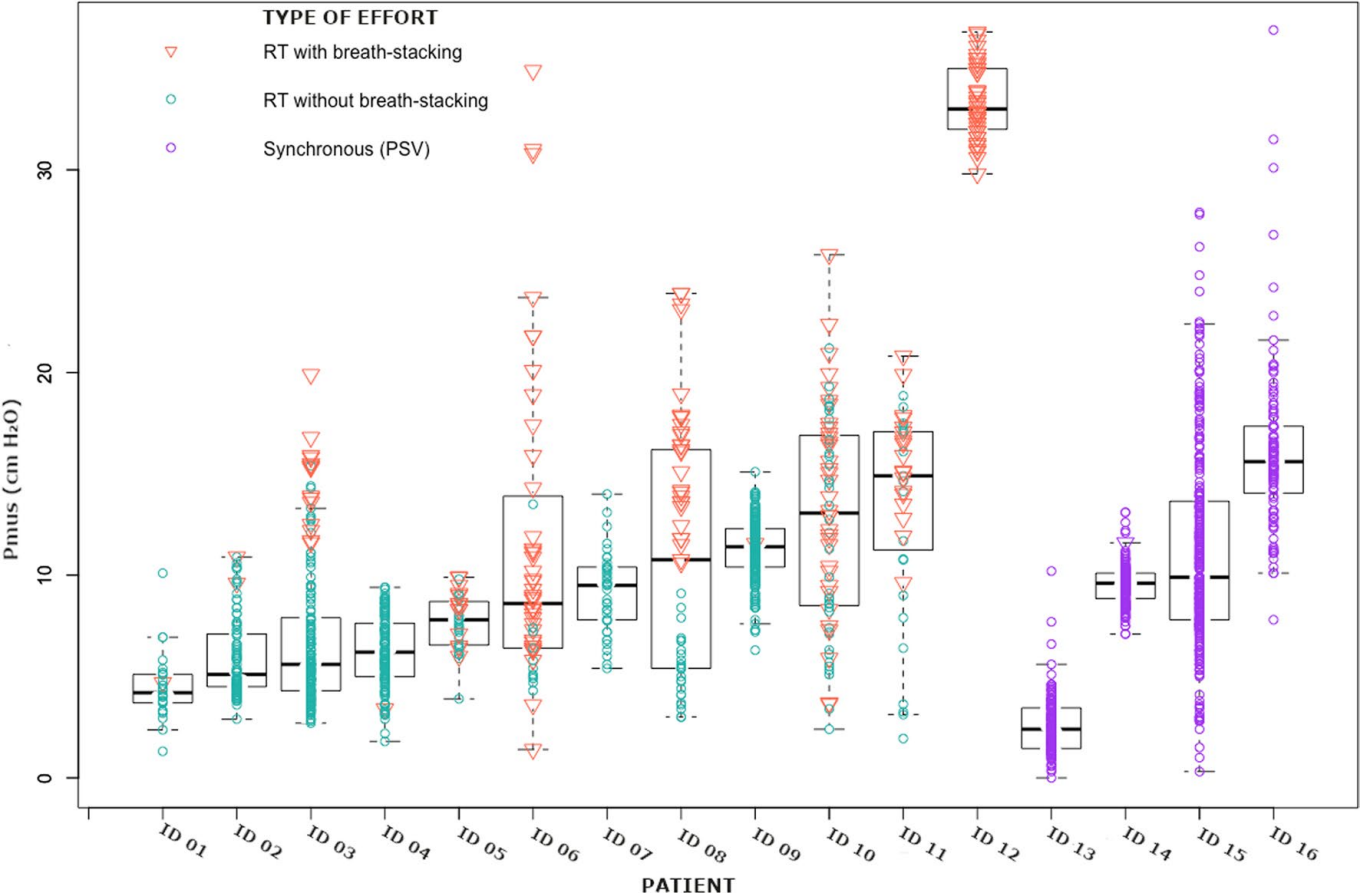
Variability and distribution of the amplitude of inspiratory effort for each patient (indicated by ID number). Amplitude of Pmus calculated for each breath during RT and during patient-triggered synchronous efforts on pressure support ventilation for each patient. RT with BS are represented with orange triangles, RT without BS are seen in green circles, and those synchronous patient-triggered efforts with violet circles

To evaluate the range of efforts generated by RT together with its variability, the muscular pressure (Pmus) was calculated for each breath in 1047 breaths with RT during assist-control ventilation (5 in volume control, 7 in pressure control) and was compared to 715 breaths from 4 tracings during pressure support ventilation including 33 expiratory ineffective efforts occurring within triggered breaths. RT efforts were separated into those inducing breath stacking (n = 206) and those without breath stacking (n = 841). The results are presented in Fig. 3. The median [25th–75th percentiles] of Pmus for RT was 8.7 [5.6;9.9] cmH_2_0, ranging from 1.3 to 36.8 cmH_2_0. Between- and within-patient variability in RT effort was considerable with median values ranging from 4.2 to 33.0 cm H_2_O and within-patient coefficient of variabilities ranging from 5 to 106% (Fig. 4). RT with breath stacking corresponded to the highest levels of Pmus, while breaths with RT but no breath stacking and triggered breaths during pressure support were of similar magnitude for Pmus. Ineffective efforts during pressure support corresponded to the lowest Pmus. Within each tracing, the median rate of RT was 5.5 [4.0;11.0] per minute (vs 16.6 [11.0, 20.3] for patient-triggered breaths) and the corresponding product of the amplitude of RT and rate (Pmus x rate of RT) was 67.3 [49.2;109.0] cmH_2_O min^−1^ (vs 172.7 [135.5, 213.2] cmH_2_O min^−1^ for patient-triggered breath).

## Discussion

We showed that an automated machine learning detection using only airway pressure and flow tracings was able to diagnose reverse triggering with a high specificity, positive and negative predictive values resulting in an excellent accuracy. Despite some false negatives, sensitivity remained very good and specificity was strong. In this selected set of tracings, RT was present in 23% of the breaths and generated a median muscle pressure of 9 cmH_2_O with important variability between and within patients ranging from 1 to 36 cmH_2_O.

### Accuracy of the algorithm

RT is a phenomenon that has been described recently and is very likely underdiagnosed at the bedside [6, 30]. Visual detection of dyssynchrony is impractical and challenging due to clinicians’ lack of time and/or expertise, and there is an urgent need for reliable automatic detection. We reasoned that any diagnostic tool implemented for daily practice would require an algorithm that diagnoses mostly impactful events, i.e., with potential clinical consequences, while avoiding false positives that would impair interpretation. The algorithms validated in this study fulfill these major conditions by displaying a very strong specificity (when a RT is detected, it is actually a RT and not a normal breath or an artifact) and a high sensitivity (most RTs are detected). The false negatives found in this study were reviewed and corresponded to weak efforts close to the limits for detection. Modifying the threshold for detection of these weak efforts could reclassify these breaths as RT but could increase the risk of false positive that really needs to be avoided. PPV and NPV had very high values in our study but are dependent on the prevalence of the event (The higher the prevalence, the higher the PPV and the lower the NPV and vice versa). The rate of RT in unselected invasively ventilated patients is still unclear, but recent reports mentioned high incidence between 25 and 50% (manuscript in revision). Use of these automatic detection algorithms will help obtaining a precise quantification of this likely underestimated phenomenon.

### Continuous assessment and quantification

A major asset of the technique is the possibility to quantify RT continuously with no additional catheter or invasive device. Paw and Flow are readily available on all ventilators, and the system can communicate and capture data from all ventilators for online analysis or subsequent review. Continuous assessment of RT is relevant to identify the changes along the patients’ course of mechanical ventilation and to quantify clusters of dyssynchrony that might have an impact on the outcome. It would also allow to obtain reliable epidemiologic data and to examine relationship between RT and treatments or relationship with other biological phenomena in a continuous manner. A few other studies have previously evaluated the accuracy of automatic detections of some types of dyssynchronies [31–34] but only one evaluated RT [35]. The latter found excellent algorithm performance but only evaluated patients with volume-controlled ventilation for ARDS during a very short period (20 consecutive breaths per patient) with only 27% of the breaths of this validation study having Pes available for the evaluation by a single expert. For the remaining breaths (72.6%) evaluated by a group of 7 experts, they did not reach complete agreement in 27% of the breaths and did not have access to Pes or EAdi signals to confirm their classification.

### Efforts generated during RT

This is the first study providing a quantification of the muscular efforts exerted during different forms of RT and compared to triggered breaths and ineffective efforts. We identified a broad range of efforts from almost negligible to huge contractions showing considerable between- and within-patient variability. Their impact on lung and diaphragm may therefore vary considerably. Physiological [36–38] and epidemiological [39] data showed that the intensity of breathing effort has variable consequences on the lung by generating varying levels of stress and strain, and on the diaphragm by modifying the risk of disuse atrophy versus load-induced injury. In these randomly selected tracings, the amplitude of effort during RT without breath stacking occurring during assist-control modes was similar to that occurring during synchronous efforts on pressure support. Interestingly, despite the levels of breathing effort during RT being considerable, they are often missed by the majority of clinicians relying on standard monitoring techniques to detect the presence of breathing effort (e.g., total rate on the ventilator equals the set rate). As expected, RT associated with breath stacking corresponded to higher levels of efforts, therefore being potentially the most injurious for the lung and the diaphragm. Additionally, the timing of RT within the respiratory cycle (i.e., occurring during inspiration or expiration) might also lead to specific mechanisms of injury such as eccentric diaphragmatic contractions or pendelluft [14, 40]. Other important aspects of breathing effort during RT might also have an impact on the lung and the diaphragm such as duration and the overall frequency of the events. Results from future study (e.g., BEARDS) are needed to provide additional information regarding outcome.

### Limitations

Our main goal was to evaluate the accuracy of the automatic detection on different types of tracings in terms of modes and patient’s effort, and we assessed a large number of breaths but in a relatively limited number of patients. Our tracing selection included RT rates ranging from 0 to 93.3% representing a broad spectrum of clinical situations. We evaluated pressure control, volume control and pressure support ventilation, which are the most frequently used modes worldwide [41, 42]. We cannot extrapolate the algorithm performances to other modes. We arbitrarily limited our definition of RT to patients’ efforts that reached maximum intensity within 1.5 s after the start of mandatory breath to differentiate RT from ineffective effort during expiration. Some patients have a fluctuating respiratory drive and intermittently trigger the ventilator. Reverse-triggered breaths usually keep the same characteristics of phase lag. We used a simple operational definition for RT not taking into account entrainment pattern. Entrainment patterns might result from different underlying causes and mechanisms. Validation of an automatic detection of this dyssynchrony is the first step to better describe RT and grasp the full spectrum of its physiologic mechanism, consequences and prevention.

## Conclusion

An algorithm for automatic detection for reverse triggering showed excellent performances with usual modes of MV. This monitoring tool allows accurate continuous assessment and diagnosis of RT which is the first step to better understand this phenomenon underlying physiologic mechanism, assess its impact and ultimately propose treatment to prevent clinical complications.

## Supplementary Information

**Additional file 1.** Online Supplement.

## Data Availability

Data and material can be shared on request.

## Abbreviations

EAdi: Electrical activity of the diaphragm
ICU: Intensive care unit
NPV: Negative predictive value
Paw: Airway pressure
Pes: Esophageal pressure
Pmus: Muscular pressure
PPV: Positive predictive value
RT: Reverse triggering

## Code availability

Custom code can be shared if requested.
