# Supplementary material for: Automated detection and quantification of reverse triggering effort under mechanical ventilation
Source: Crit Care. 2021 Feb 15;25:60. doi: 10.1186/s13054-020-03387-3 (PMC7883535; doi:10.1186/s13054-020-03387-3)

**Automated Detection and Quantification of Reverse Triggering Effort under Mechanical Ventilation: Online supplement**

[Identification of the initiation of a ventilator breath 2](#_Toc54562339)

[Pre-validation phase 2](#_Toc54562340)

[Description of the algorithms for RT detection (e-Fig 1) 3](#_Toc54562341)

[Time from insufflation to reverse triggering initiation 5](#_Toc54562342)

[References 5](#_Toc54562343)

[e-figure 1: example of reverse triggering during inspiratory pause 6](#_Toc54562344)

[e-Figure 2: Example of reverse triggering happening during late inspiration and the beginning of exhalation 7](#_Toc54562345)

[e-Figure 3. Example of Reverse triggering with and without breath stacking 8](#_Toc54562346)

[e-Figure 4. Tracing selections 9](#_Toc54562347)

[e-Figure 5. Receiving operator curve (ROC curve) showing the automatic algorithm performance 10](#_Toc54562348)

# Identification of the initiation of a ventilator breath

Identification of a patient- or machine-triggered breath was assessed from a portion of the Paw waveform which contained the initiation of the inspiratory phase. High frequency components were removed to avoid transient changes. The minimum Paw value within that portion was compared to a baseline. If the minimum value was lower than the baseline by more than a threshold adapted to the ventilation mode, then the breath was patient-triggered otherwise the breath was machine-triggered. Additionally, for assist-control modes, if the current expiratory time deviated by more than a 10% from the mean expiratory time over the last 10 breaths then the breath is finally labelled as patient-triggered.

# Pre-validation phase

We randomly selected tracings from two studies in which Paw, flow and either Pes or EAdi waveforms were collected in patients with invasive mechanical ventilation (DIVIP NCT03447288 and BEARDS NCT02434016). Breaths were reviewed, discussed and labelled through biweekly teleconference used to develop or refine algorithms based on deformation of flow and Paw (or changes in slope indicated by second derivatives of the signal). Several algorithms were necessary depending on the moment of the breath cycle at which most of the effort occurred (insufflation, plateau and expiration) and on the mode of ventilation (volume or pressure targeted). Algorithms for RT were created, tested and refined several times until they offered a good performance on the selected tracings.

In this phase, 12 tracings (9 from the DIVIP study and 3 from the BEARDS study were assessed and algorithms were fine-tuned based on videoconference discussions and output of the visual evaluation. Discrepancies were discussed and led to algorithms modification.

Pre-validation phase: Confusion Matrix

|  |  | Visual assessment | | Total |
| --- | --- | --- | --- | --- |
|  |  | YES | NO |  |
| Automatic detection | YES | 790 | 60 | 850 |
|  | NO | 119 | 2554 | 2673 |
| Total |  | 909 | 2614 | 3523 |

Based on this confusion matrix, global accuracy is 95%, Sensitivity is 87%, Specificity is 98%, Positive Predictive Value is 93% and Negative Predictive Value is 96%.

Kappa: 0.89 [0.87- 0.90]

# Description of the algorithms for RT detection (e-Fig 1)

Diverse methods of digital signal processing and supervised machine learning have been combined to detect RT events from airflow and airway pressure (Paw) waveforms. Depending on the ventilatory modality and the respiratory phase where the event occurs, it can change the waveform of either the airflow, the pressure or both, with lesser or greater level of alterations. Thus, these methods intend to account for the different ways RT events impact on the waveforms.

The methods use the ventilatory modality and the mechanism that has initiated the respiratory cycle (i.e., non-patient or patient triggered) as inputs. This information is crucial to automatically select the strategy to follow on, which determines if a RT event has occurred during the inspiratory, the pause (if any), and/or the expiratory phases. Figure 1 summarizes the strategies implemented.

In volume-controlled ventilation (VCV) mode, we mainly use the airway pressure waveform to detect the presence of an RT event during inspiration, pause or expiration, by separately. RT occurring early enough in the inspiratory phase are detected from the smoothed Paw waveform based on a differentiation procedure to look for zero-crossings, and/or if the actual peak pressure (Ppeak) value is decreased by more than a dynamically updated tolerance computed from the most immediate previous Ppeak values, to track changes in its values. During pause (if any), the algorithm takes into account the absolute value of ∆Ppeak and the absolute variation (also to the average of its immediate previous values) of the difference of the plateau pressure (Pplat) and the Ppeak value, ∆P, to account for both the decreased Ppeak and the loss of plateau due to the patient’s effort observed in such cases (e-Fig. 2). Then, a logistic equation with the above two measures as input variables is used to create a regression model which informs about the presence of RT events with a certain probability value. RT occurring late at the inspiration, provoking a decreased peak expiratory flow (PEF) and slow rate of change at the onset of exhalation (e-Fig. 3), are detected by using another logistic regression model with the PEF and the slope of the onset of exhalation till the point of the PEF in flow waveform as input variables.

In pressure-controlled ventilation (PCV) or in auto-triggered breaths in pressure support (PS) mode, methods mainly work on the airflow waveform. Detection of RT events early enough in the inspiration phase is based on the identification of significant zero-crossings in the differentiated output of the smoothed airflow. If the zero-crossing is not clear enough then the algorithm looks for a slight drop in Paw starting at the point when the presumable relevant zero-crossing is identified in the differentiated output of the smoothed airflow.

Independently of the ventilatory mode, RT events occurring during the expiratory phase are detected if the actual expiratory flow curve deviates (0%=almost inexistent, 100%=clearly event) from the theoretical mono-exponential one, similarly as was previously developed for IEE detection [1], with the added time constraint that the patient’s effort can only occur within the interval of 1.5 seconds after the controlled inspiration is initiated. When the patient’s effort is strong enough to trigger a second (breath-stacked) breath, it is detected by using some mathematical calculations which take into account differences in timing and the amount of air volume between inspiration and expiration phases, similarly as was previously published [2].

# **Time from insufflation to reverse triggering initiation**

We assessed the delay from the beginning of the passive insufflation to the initiation of the reverse-triggering to identify the impact of this lag on the development of breath stacking.

We examined the tracings from 6 patients who had RT both with frequent and without breath-stacking and compared the delay between the start of insufflation and the start of RT effort for the two types of RTs. The delay was significantly longer for breath stacked RT effort than for RT without breath stacking in two patients, was not different in two patients and was significantly shorter for two patients.

# References

1. Blanch L, Sales B, Montanya J, et al (2012) Validation of the Better Care® system to detect ineffective efforts during expiration in mechanically ventilated patients: a pilot study. Intensive Care Med 38:772–780. https://doi.org/10.1007/s00134-012-2493-4

2. de Haro C, López-Aguilar J, Magrans R, et al (2018) Double Cycling During Mechanical Ventilation: Frequency, Mechanisms, and Physiologic Implications. Crit Care Med 46:1385–1392. https://doi.org/10.1097/CCM.0000000000003256

# e-figure 1: example of reverse triggering during inspiratory pause


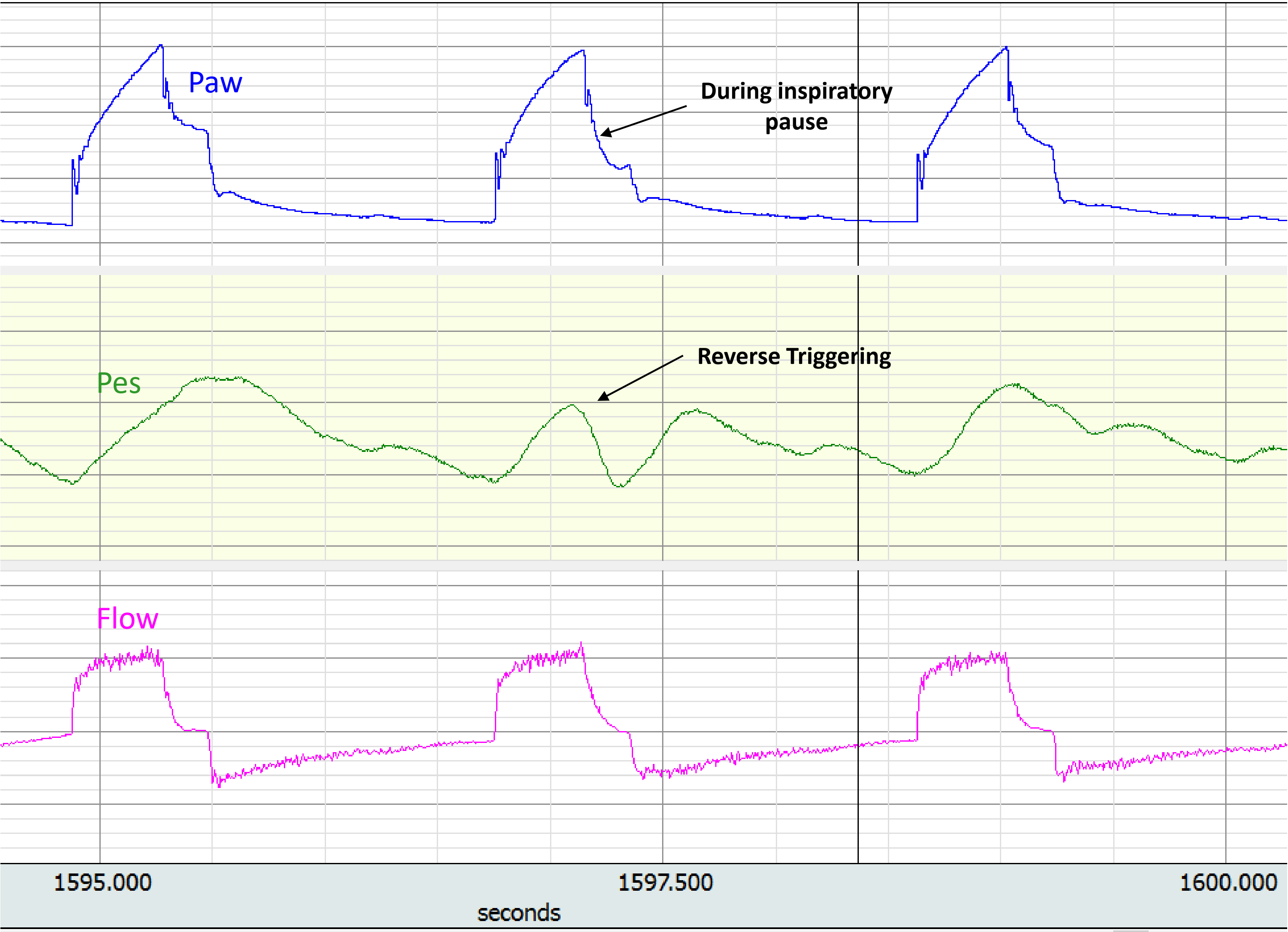


# e-Figure 2: Example of reverse triggering happening during late inspiration and the beginning of exhalation


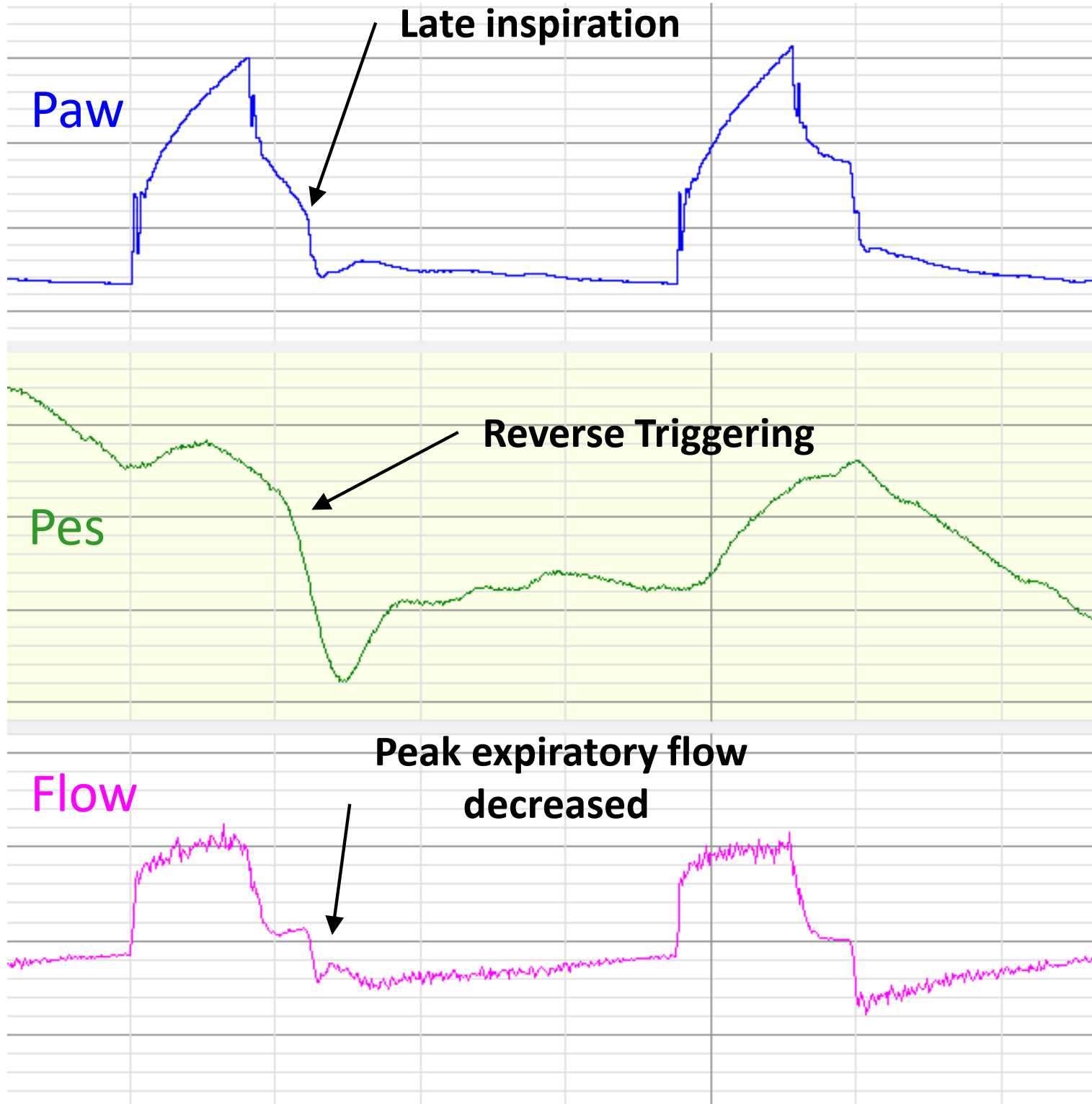


# e-Figure 3. Example of Reverse triggering with and without breath stacking

#


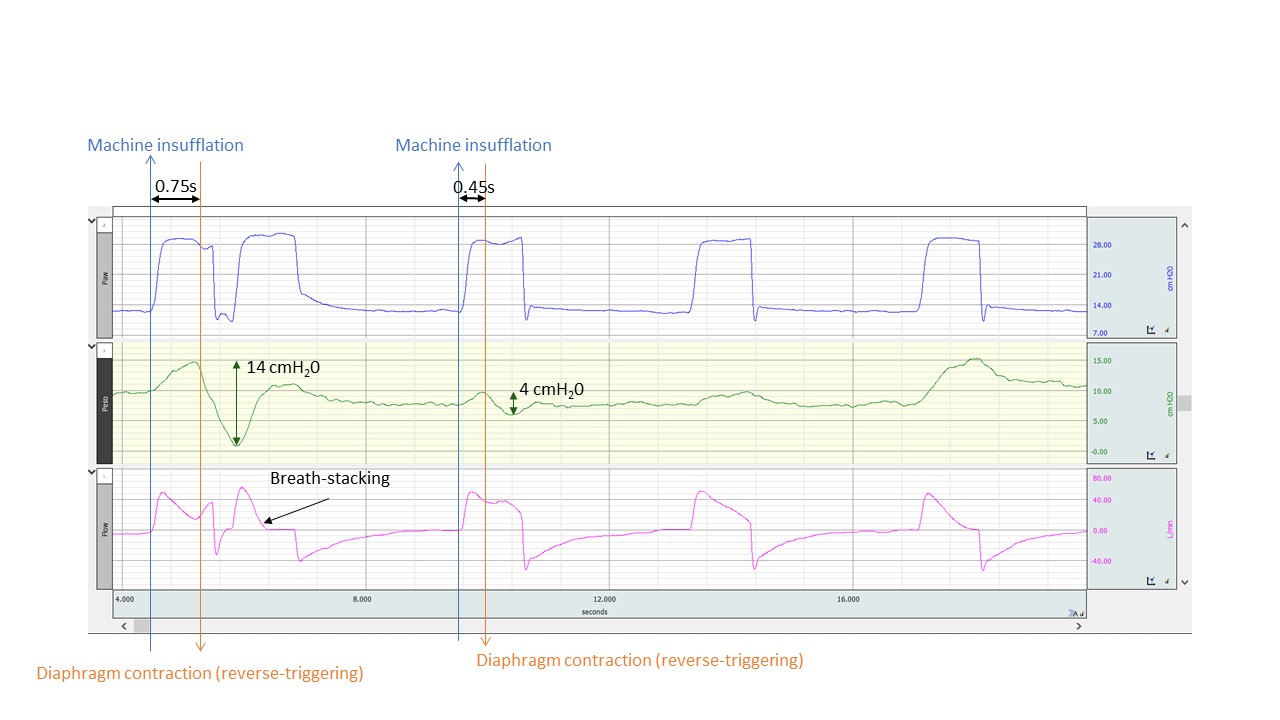


# e-Figure 4. Tracing selections

A total of 1087 tracings were available at the moment of the study. Visual assessment allowed to classify the tracings in 5 groups (paralyzed, few/some/many Reverse-triggering and PSV). Four tracings were randomly selected in each group.

**Abbreviations: PAC, pressure assist control; PSV, pressure support ventilation; RT, reverse triggering ; VAC, volume assist controlled**

# e-Figure 5. Receiving operator curve (ROC curve) showing the automatic algorithm performance


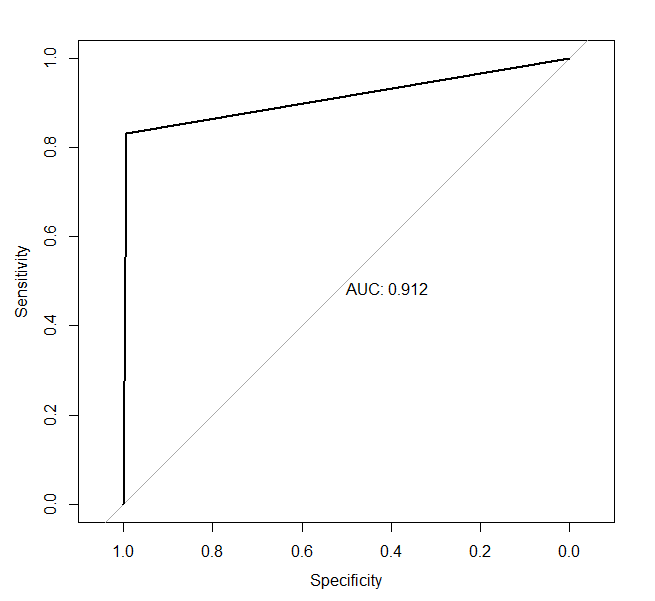

Supplement: Supplementary file 1 — Additional file 1. Online Supplement. [file 13054_2020_3387_MOESM1_ESM.docx]
